# 2-Hydr­oxy-1-methoxy­anthraquinone monohydrate

**DOI:** 10.1107/S1600536809021254

**Published:** 2009-06-06

**Authors:** Zhi-Meng Liu, Yuan-Qi Jiao

**Affiliations:** a School of Materials Science and Engineering, South China University of Technology, Guangzhou 510641, People’s Republic of China

## Abstract

The title compound, C_15_H_10_O_4_·H_2_O, also known as alizarin 1-methyl ether monohydrate, was isolated from *Morinda officinalis* How. The anthraquinone ring system is almost planar, the dihedral angle between the two outer benzene rings being 3.07 (4)°. In the crystal structure, O—H⋯O hydrogen bonds link the organic mol­ecules and the water mol­ecules, forming a three-dimensional network.

## Related literature

For pharmacological properties of anthraquinone derivatives, see: Kim *et al.* (2005 ▶) and of 1-meth­oxy-2-hydroxy­anthraquinone, see: Ali *et al.* (2000 ▶); Jia *et al.* (2007 ▶); Wu *et al.* (2003 ▶). For related structures, see: Boonnak *et al.* (2005 ▶); Ng *et al.* (2005 ▶). For the structure of another compound isolated from *Morinda officinalis* How., see: Xu *et al.* (2009 ▶). For reference structural data, see: Allen *et al.* (1987 ▶).
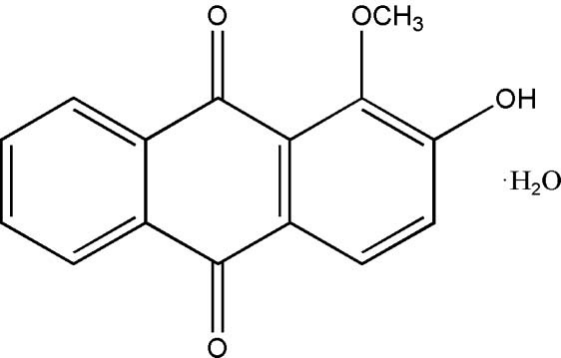


## Experimental

### 

#### Crystal data

C_15_H_10_O_4_·H_2_O
*M* *_r_* = 272.25
Triclinic, 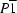

*a* = 7.9583 (19) Å
*b* = 8.269 (2) Å
*c* = 10.188 (2) Å
α = 102.462 (3)°
β = 102.364 (3)°
γ = 100.653 (3)°
*V* = 620.4 (2) Å^3^
*Z* = 2
Mo *K*α radiation
μ = 0.11 mm^−1^
*T* = 298 K
0.30 × 0.20 × 0.15 mm

#### Data collection

Bruker APEXII area-detector diffractometer
Absorption correction: multi-scan (*SADABS*; Sheldrick, 1996 ▶) *T*_min_ = 0.973, *T*_max_ = 0.986
3218 measured reflections
2198 independent reflections
1488 reflections with *I* > 2σ(*I*)
*R*_int_ = 0.013

#### Refinement

*R*[*F* ^2^ > 2σ(*F* ^2^)] = 0.046
*wR*(*F* ^2^) = 0.147
*S* = 1.04
2198 reflections
191 parameters
3 restraints
H atoms treated by a mixture of independent and constrained refinement
Δρ_max_ = 0.27 e Å^−3^
Δρ_min_ = −0.17 e Å^−3^

### 

Data collection: *APEX2* (Bruker, 2004 ▶); cell refinement: *SAINT* (Bruker, 2004 ▶); data reduction: *SAINT*; program(s) used to solve structure: *SHELXS97* (Sheldrick, 2008 ▶); program(s) used to refine structure: *SHELXL97* (Sheldrick, 2008 ▶); molecular graphics: *SHELXTL* (Sheldrick, 2008 ▶); software used to prepare material for publication: *SHELXL97*.

## Supplementary Material

Crystal structure: contains datablocks I, global. DOI: 10.1107/S1600536809021254/bg2258sup1.cif

Structure factors: contains datablocks I. DOI: 10.1107/S1600536809021254/bg2258Isup2.hkl

Additional supplementary materials:  crystallographic information; 3D view; checkCIF report

## Figures and Tables

**Table 1:** Hydrogen-bond geometry (Å, °)

*D*—H⋯*A*	*D*—H	H⋯*A*	*D*⋯*A*	*D*—H⋯*A*
O1*W*—H1*W*⋯O2^i^	0.86 (3)	2.31 (2)	2.960 (3)	133 (3)
O1*W*—H2*W*⋯O4^ii^	0.87 (3)	2.30 (2)	3.072 (3)	149 (3)
O3—H3⋯O1*W*	0.82	1.87	2.687 (2)	173

Symmetry codes: (i) 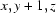; (ii) 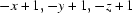.

**Fig. 1.**
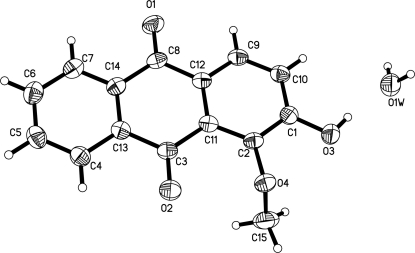
The molecular structure showing the atomic-numbering scheme and displacement ellipsoids drawn at the 30% probability level.

**Fig. 2.**
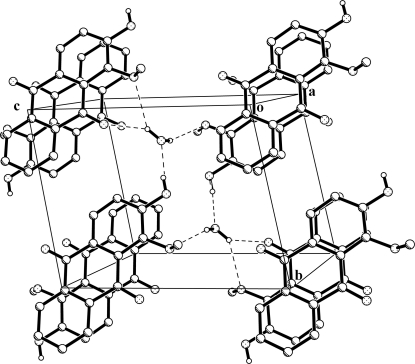
The molecular packing showing the hydrogen bonding interactions as broken lines.

## supplementary crystallographic information

### Comment

Anthraquinone derivatives extracted from the roots of Morinda officinalis How (most common familiar name in China: Bajitian) have been used in China since ancient times to treat a wide range of symptoms including poor digestion, high blood pressure and immune deficiencies . Recent studies have demonstrated that they have multiple pharmacological actions (Kim *et al.*, 2005). One component found in Morinda officinalis How, 1-Methoxy-2-hydroxyanthraquinone, is known as alizarin-1-methylether and exhibits a variety of potent biological effects such as antiviral and antimicrobial activities (Ali *et al.*, 2000), antioxidant activity (Jia *et al.*, 2007) and cyototoxic activity (Wu *et al.*, 2003). We report here the structure of the monhydrate.

In the title compound (Fig. 1), the C-C bond lengths show normal values (Allen *et al.*, 1987), and the C-O and C=O bond lengths are comparable to those observed in similar structures (Ng *et al.*, 2005; Boonnak *et al.*, 2005). The anthraquinone ring system is substantially planar, the dihedral angle between the two benzene rings being 3.07 (4)°. In the crystal structure, the crystal water connects with alizarin-1-methylether by O—H···.O hydrogen bonds.The molecules are self-assembled by O—H···.O hydrogen bonding interactions (Table 1 and Fig. 2) into a supramolecular network.

### Experimental

The roots of Morinda officinalis How (1000 g) were shattered to powder (about 30 mesh) and extracted with 85% ethanol (4000 ml) for 2 h with stirring. The extraction procedure was repeated three times. The extracts were combined and evaporated to dryness under reduced pressure at 333 K, the residue was redissolved in water (800 ml). Then the enriched extracts were extracted with chloroform three times (800 ml for each time), the chlorofrom solution were combined and evaporated to dryness under reduced pressure at 333 K. 6.80 g of crude extracts were obtained. The crude extracts were separated with n-hexane-ethyl acetate-methanol-water (6 : 4 : 5 : 5, v/v) using high-speed counter-current chromatography (HSCCC) to obtain 1-Methoxy-2-hydroxyanthraquinone (yield 90.6 mg). Single crystals suitable for X-ray analysis were obtained by slow evaporation of a methanol solution.

### Refinement

H atoms not pertaining to water molecules were placed at calculated positions and treated as riding on the parent atoms with C—H = 0.93–0.97 and O—H = 0.82 Å, and with *U*_iso_(H) = 1.2 or 1.5 *U*_eq_(C, O). The water hydrogen atoms were found from the Fourier maps and refined with restrained O-H=0.86 (3)Å and free *U*_iso_(H).

### Crystal data

**Table:**

C_15_H_10_O_4_·H_2_O	*Z* = 2
*M**_r_* = 272.25	*F*(000) = 284.0
Triclinic, *P*1	*D*_x_ = 1.457 Mg m^−^^3^
Hall symbol: -P 1	Mo *K*α radiation, λ = 0.71073 Å
*a* = 7.9583 (19) Å	Cell parameters from 1018 reflections
*b* = 8.269 (2) Å	θ = 2.6–26.2°
*c* = 10.188 (2) Å	µ = 0.11 mm^−^^1^
α = 102.462 (3)°	*T* = 298 K
β = 102.364 (3)°	Block, yellow
γ = 100.653 (3)°	0.30 × 0.20 × 0.15 mm
*V* = 620.4 (2) Å^3^	

### Data collection

**Table:**

Bruker APEXII area-detector diffractometer	2198 independent reflections
Radiation source: fine-focus sealed tube	1488 reflections with *I* > 2σ(*I*)
graphite	*R*_int_ = 0.013
φ and ω scans	θ_max_ = 25.2°, θ_min_ = 2.1°
Absorption correction: multi-scan (*SADABS*; Sheldrick, 1996)	*h* = −9→9
*T*_min_ = 0.973, *T*_max_ = 0.986	*k* = −9→9
3218 measured reflections	*l* = −12→9

### Refinement

**Table:**

Refinement on *F*^2^	Primary atom site location: structure-invariant direct methods
Least-squares matrix: full	Secondary atom site location: difference Fourier map
*R*[*F*^2^ > 2σ(*F*^2^)] = 0.046	Hydrogen site location: inferred from neighbouring sites
*wR*(*F*^2^) = 0.147	H atoms treated by a mixture of independent and constrained refinement
*S* = 1.04	*w* = 1/[σ^2^(*F*_o_^2^) + (0.0778*P*)^2^ + 0.0805*P*] where *P* = (*F*_o_^2^ + 2*F*_c_^2^)/3
2198 reflections	(Δ/σ)_max_ < 0.001
191 parameters	Δρ_max_ = 0.27 e Å^−^^3^
3 restraints	Δρ_min_ = −0.17 e Å^−^^3^

### Special details

**Table:**

Geometry. All e.s.d.'s (except the e.s.d. in the dihedral angle between two l.s. planes) are estimated using the full covariance matrix. The cell e.s.d.'s are taken into account individually in the estimation of e.s.d.'s in distances, angles and torsion angles; correlations between e.s.d.'s in cell parameters are only used when they are defined by crystal symmetry. An approximate (isotropic) treatment of cell e.s.d.'s is used for estimating e.s.d.'s involving l.s. planes.
Refinement. Refinement of *F*^2^ against ALL reflections. The weighted *R*-factor *wR* and goodness of fit *S* are based on *F*^2^, conventional *R*-factors *R* are based on *F*, with *F* set to zero for negative *F*^2^. The threshold expression of *F*^2^ > σ(*F*^2^) is used only for calculating *R*-factors(gt) *etc*. and is not relevant to the choice of reflections for refinement. *R*-factors based on *F*^2^ are statistically about twice as large as those based on *F*, and *R*- factors based on ALL data will be even larger.

### Fractional atomic coordinates and isotropic or equivalent isotropic displacement parameters (Å^2^)

**Table:**

	*x*	*y*	*z*	*U*_iso_*/*U*_eq_	
C1	0.6640 (3)	0.3815 (2)	0.3121 (2)	0.0509 (5)	
C2	0.7049 (3)	0.2214 (2)	0.30375 (19)	0.0476 (5)	
C3	0.7720 (3)	−0.0406 (2)	0.1655 (2)	0.0485 (5)	
C4	0.8497 (3)	−0.2806 (2)	0.0176 (2)	0.0555 (5)	
H4	0.8772	−0.3219	0.0955	0.067*	
C5	0.8632 (3)	−0.3695 (3)	−0.1086 (3)	0.0645 (6)	
H5	0.8980	−0.4717	−0.1158	0.077*	
C6	0.8257 (3)	−0.3093 (3)	−0.2248 (3)	0.0668 (6)	
H6	0.8347	−0.3707	−0.3099	0.080*	
C7	0.7746 (3)	−0.1565 (3)	−0.2142 (2)	0.0600 (6)	
H7	0.7505	−0.1146	−0.2921	0.072*	
C8	0.7044 (3)	0.0971 (2)	−0.07708 (19)	0.0500 (5)	
C9	0.6444 (3)	0.3450 (2)	0.0710 (2)	0.0514 (5)	
H9	0.6210	0.3853	−0.0078	0.062*	
C10	0.6335 (3)	0.4407 (3)	0.1954 (2)	0.0542 (5)	
H10	0.6054	0.5458	0.2006	0.065*	
C11	0.7216 (2)	0.1254 (2)	0.17820 (19)	0.0438 (5)	
C12	0.6896 (2)	0.1890 (2)	0.06001 (19)	0.0444 (5)	
C13	0.7951 (2)	−0.1285 (2)	0.0293 (2)	0.0462 (5)	
C14	0.7593 (2)	−0.0664 (2)	−0.08792 (19)	0.0472 (5)	
C15	0.8905 (4)	0.2159 (3)	0.5181 (2)	0.0869 (8)	
H15A	0.9726	0.1661	0.4763	0.130*	
H15B	0.8843	0.1791	0.6005	0.130*	
H15C	0.9298	0.3380	0.5425	0.130*	
O1	0.6741 (2)	0.15409 (19)	−0.17812 (15)	0.0741 (5)	
O2	0.7941 (3)	−0.10671 (19)	0.26171 (16)	0.0772 (5)	
O3	0.6543 (2)	0.46790 (18)	0.43714 (14)	0.0679 (5)	
H3	0.6315	0.5594	0.4317	0.102*	
O4	0.7180 (2)	0.16259 (18)	0.42088 (14)	0.0616 (4)	
O1W	0.5800 (3)	0.7728 (2)	0.44181 (19)	0.0829 (6)	
H1W	0.597 (4)	0.844 (4)	0.393 (4)	0.162 (15)*	
H2W	0.472 (2)	0.762 (4)	0.450 (4)	0.134 (14)*	

### Atomic displacement parameters (Å^2^)

**Table:**

	*U*^11^	*U*^22^	*U*^33^	*U*^12^	*U*^13^	*U*^23^
C1	0.0497 (12)	0.0517 (11)	0.0508 (11)	0.0118 (9)	0.0133 (9)	0.0132 (9)
C2	0.0446 (11)	0.0537 (11)	0.0457 (11)	0.0084 (9)	0.0097 (8)	0.0211 (9)
C3	0.0464 (11)	0.0500 (11)	0.0492 (11)	0.0071 (9)	0.0100 (9)	0.0196 (9)
C4	0.0493 (12)	0.0548 (12)	0.0635 (13)	0.0132 (9)	0.0144 (10)	0.0182 (10)
C5	0.0549 (14)	0.0588 (13)	0.0799 (16)	0.0187 (10)	0.0190 (11)	0.0134 (11)
C6	0.0577 (14)	0.0715 (15)	0.0633 (14)	0.0117 (12)	0.0181 (11)	0.0030 (11)
C7	0.0561 (13)	0.0671 (14)	0.0510 (12)	0.0073 (11)	0.0120 (10)	0.0128 (10)
C8	0.0450 (11)	0.0557 (11)	0.0432 (11)	0.0020 (9)	0.0041 (8)	0.0166 (9)
C9	0.0515 (12)	0.0506 (11)	0.0516 (11)	0.0082 (9)	0.0067 (9)	0.0226 (9)
C10	0.0557 (13)	0.0504 (11)	0.0565 (12)	0.0133 (9)	0.0096 (10)	0.0191 (10)
C11	0.0370 (10)	0.0449 (10)	0.0490 (11)	0.0051 (8)	0.0090 (8)	0.0179 (8)
C12	0.0384 (10)	0.0451 (10)	0.0462 (11)	0.0028 (8)	0.0060 (8)	0.0163 (8)
C13	0.0360 (10)	0.0464 (11)	0.0533 (11)	0.0039 (8)	0.0094 (8)	0.0146 (9)
C14	0.0382 (10)	0.0504 (11)	0.0468 (11)	0.0010 (8)	0.0082 (8)	0.0115 (8)
C15	0.100 (2)	0.0976 (19)	0.0591 (15)	0.0295 (16)	−0.0015 (14)	0.0303 (14)
O1	0.1049 (13)	0.0732 (10)	0.0481 (9)	0.0260 (9)	0.0141 (8)	0.0263 (7)
O2	0.1226 (15)	0.0696 (10)	0.0615 (10)	0.0433 (10)	0.0352 (9)	0.0355 (8)
O3	0.0926 (12)	0.0649 (10)	0.0561 (9)	0.0349 (9)	0.0257 (8)	0.0172 (7)
O4	0.0760 (10)	0.0684 (9)	0.0503 (8)	0.0229 (8)	0.0207 (7)	0.0277 (7)
O1W	0.1221 (18)	0.0768 (12)	0.0776 (12)	0.0454 (11)	0.0478 (11)	0.0384 (10)

### Geometric parameters (Å, °)

**Table:**

C1—O3	1.345 (2)	C8—O1	1.218 (2)
C1—C10	1.373 (3)	C8—C12	1.479 (3)
C1—C2	1.410 (3)	C8—C14	1.486 (3)
C2—O4	1.374 (2)	C9—C10	1.371 (3)
C2—C11	1.399 (3)	C9—C12	1.390 (3)
C3—O2	1.218 (2)	C9—H9	0.9300
C3—C13	1.487 (3)	C10—H10	0.9300
C3—C11	1.487 (3)	C11—C12	1.408 (2)
C4—C5	1.373 (3)	C13—C14	1.395 (3)
C4—C13	1.394 (3)	C15—O4	1.437 (3)
C4—H4	0.9300	C15—H15A	0.9600
C5—C6	1.377 (3)	C15—H15B	0.9600
C5—H5	0.9300	C15—H15C	0.9600
C6—C7	1.387 (3)	O3—H3	0.8200
C6—H6	0.9300	O1W—H1W	0.86 (3)
C7—C14	1.382 (3)	O1W—H2W	0.87 (3)
C7—H7	0.9300		
			
O3—C1—C10	123.24 (18)	C12—C9—H9	119.2
O3—C1—C2	116.77 (17)	C9—C10—C1	120.01 (18)
C10—C1—C2	119.98 (18)	C9—C10—H10	120.0
O4—C2—C11	122.55 (17)	C1—C10—H10	120.0
O4—C2—C1	117.08 (17)	C2—C11—C12	118.56 (17)
C11—C2—C1	120.29 (16)	C2—C11—C3	122.38 (16)
O2—C3—C13	119.21 (18)	C12—C11—C3	119.06 (17)
O2—C3—C11	122.56 (18)	C9—C12—C11	119.58 (18)
C13—C3—C11	118.22 (16)	C9—C12—C8	117.85 (17)
C5—C4—C13	120.20 (19)	C11—C12—C8	122.57 (17)
C5—C4—H4	119.9	C4—C13—C14	118.95 (18)
C13—C4—H4	119.9	C4—C13—C3	118.85 (17)
C4—C5—C6	120.9 (2)	C14—C13—C3	122.18 (17)
C4—C5—H5	119.6	C7—C14—C13	120.27 (19)
C6—C5—H5	119.6	C7—C14—C8	119.88 (18)
C5—C6—C7	119.6 (2)	C13—C14—C8	119.85 (18)
C5—C6—H6	120.2	O4—C15—H15A	109.5
C7—C6—H6	120.2	O4—C15—H15B	109.5
C14—C7—C6	120.1 (2)	H15A—C15—H15B	109.5
C14—C7—H7	120.0	O4—C15—H15C	109.5
C6—C7—H7	119.9	H15A—C15—H15C	109.5
O1—C8—C12	121.26 (19)	H15B—C15—H15C	109.5
O1—C8—C14	120.74 (18)	C1—O3—H3	109.5
C12—C8—C14	117.99 (16)	C2—O4—C15	115.30 (16)
C10—C9—C12	121.52 (18)	H1W—O1W—H2W	107.3 (16)
C10—C9—H9	119.2		
			
O3—C1—C2—O4	4.7 (3)	C3—C11—C12—C8	−0.1 (3)
C10—C1—C2—O4	−174.23 (17)	O1—C8—C12—C9	−1.6 (3)
O3—C1—C2—C11	−178.49 (17)	C14—C8—C12—C9	177.72 (16)
C10—C1—C2—C11	2.6 (3)	O1—C8—C12—C11	179.19 (17)
C13—C4—C5—C6	1.0 (3)	C14—C8—C12—C11	−1.5 (3)
C4—C5—C6—C7	0.3 (3)	C5—C4—C13—C14	−1.7 (3)
C5—C6—C7—C14	−0.7 (3)	C5—C4—C13—C3	176.53 (17)
C12—C9—C10—C1	−1.3 (3)	O2—C3—C13—C4	−3.0 (3)
O3—C1—C10—C9	−179.48 (18)	C11—C3—C13—C4	177.29 (16)
C2—C1—C10—C9	−0.6 (3)	O2—C3—C13—C14	175.24 (18)
O4—C2—C11—C12	174.08 (16)	C11—C3—C13—C14	−4.5 (3)
C1—C2—C11—C12	−2.5 (3)	C6—C7—C14—C13	−0.1 (3)
O4—C2—C11—C3	−6.0 (3)	C6—C7—C14—C8	−179.97 (18)
C1—C2—C11—C3	177.34 (16)	C4—C13—C14—C7	1.3 (3)
O2—C3—C11—C2	3.3 (3)	C3—C13—C14—C7	−176.93 (17)
C13—C3—C11—C2	−176.94 (16)	C4—C13—C14—C8	−178.80 (17)
O2—C3—C11—C12	−176.78 (18)	C3—C13—C14—C8	3.0 (3)
C13—C3—C11—C12	2.9 (3)	O1—C8—C14—C7	−0.7 (3)
C10—C9—C12—C11	1.3 (3)	C12—C8—C14—C7	179.92 (16)
C10—C9—C12—C8	−177.90 (17)	O1—C8—C14—C13	179.34 (18)
C2—C11—C12—C9	0.6 (3)	C12—C8—C14—C13	0.0 (3)
C3—C11—C12—C9	−179.25 (16)	C11—C2—O4—C15	95.3 (2)
C2—C11—C12—C8	179.82 (16)	C1—C2—O4—C15	−87.9 (2)

### Hydrogen-bond geometry (Å, °)

**Table:**

*D*—H···*A*	*D*—H	H···*A*	*D*···*A*	*D*—H···*A*
O1W—H1W···O2^i^	0.86 (3)	2.31 (2)	2.960 (3)	133 (3)
O1W—H2W···O4^ii^	0.87 (3)	2.30 (2)	3.072 (3)	149 (3)
O3—H3···O1W	0.82	1.87	2.687 (2)	173

Symmetry codes: (i) *x*, *y*+1, *z*; (ii) −*x*+1, −*y*+1, −*z*+1.
